# Job insecurity, emotional exhaustion, and workplace deviance: The role of corporate social responsibility

**DOI:** 10.3389/fpubh.2022.1000628

**Published:** 2022-10-06

**Authors:** Xingping Jia, Shudi Liao, Wenjun Yin

**Affiliations:** ^1^Hubei University, Wuhan, China; ^2^Hubei Center for Studies of Human Capital Development Strategy and Policy, Key Research Base of Humanities and Social Science of Hubei Province, Wuhan, China; ^3^Hubei University of Science and Technology, Xianning, China

**Keywords:** job insecurity, emotional exhaustion, employees' CSR perceptions, organizational identification, workplace deviance

## Abstract

Job insecurity is one of top concerns in the contemporary workplace, which significantly affects emotional exhaustion and workplace deviance. Thus, this study seeks to explore the buffering role of employees' corporate social responsibility (CSR) perceptions to against the effect of job insecurity. Based on micro-CSR literature and social identity theory, this study tested the proposition that employees' CSR perceptions moderate the relationship between job insecurity and emotional exhaustion through organizational identification. Using three-wave data collected from 145 employees in one of China's biggest computer equipment providers, we found that employees' CSR perceptions alleviate (exacerbate) the negative relationship between quantitative (qualitative) job insecurity and emotional exhaustion *via* organization identification. Our findings provided new insights to scholars and managers in dealing with job insecurity.

## Introduction

Faced with the economic crisis, shifting governmental policies, and the outbreak of the coronavirus (COVID-19), the whole globe has entered into the VUCA (volatility, uncertainty, complexity, and ambiguity) and TUNA (turbulent, uncertain, novel, and ambiguity) world (1). Not surprisingly, such VUCA and TUNA context bring extreme pressures to job environment, leading to employers downsizing and outsourcing labor. Job insecurity has become one of top concerns in contemporary working life (2–4).

Job insecurity reflects the degree to which employees perceive threat to the continuity and stability of employment (4). Job insecurity is a work-related stress which can bring poor mental, physical, and work-related wellbeing (e.g., anger, emotional exhaustion, disengagement, and counterproductive work behaviors), and further lead to negative work-related performance [(5); see also (6) for a review]. Accordingly, prior studies explored several ways to buffer against the adverse consequences of job insecurity such as improving leader-member exchange (LMX) (7, 8), fostering organizational justices (5) and psychological capital as well (9). While, much more attention has been paid to the direct effect of job insecurity than to buffers that against the negative effect of job insecurity (10).

In this study, we introduced corporate social responsibility (CSR) as an essential factor in minimizing the negative consequences of job insecurity. CSR refers to “the firm's considerations of, and response to, issues beyond the narrow economic, technical, and legal requirements of the firm to accomplish social (and environmental) benefits along with the traditional economic gains which the firm seeks” [(11), p. 312]. From the perspective of employees, CSR is a notable aspect of their more general justice perceptions (12), employees perceptions about a firm's CSR can overwhelming affect employees' attitudes and behaviors toward the firm (e.g., organizational identification, attachment, satisfaction, and engagement) (13–15). Further, a firm's CSR can drive certain employee attitudes and behaviors to mitigate work stress (16) and turnover intention (17). In line with these studies, therefore, we explored whether or not employees' CSR perceptions can play a buffering role in dealing with job insecurity.

**Figure 1:**
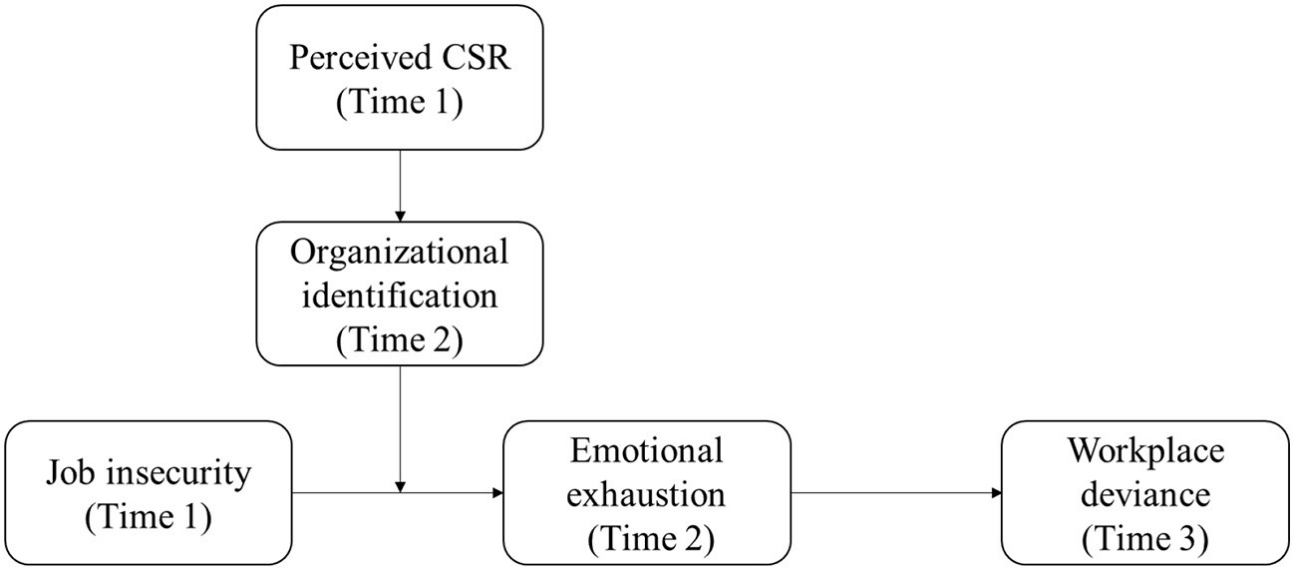
Research framework.

Based on prior study in the job insecurity literature, we developed the baseline framework about the job insecurity—emotional exhaustion—workplace deviance link. Then drawing on the micro-CSR literature and social identity theory, we explored the mechanism through which employees' CSR perceptions moderate the job insecurity and emotional exhaustion linkage. Figure 1 depicts the theoretical model of this study. We tested our model by carrying out a three-wave data collection approach.

This study makes two contributes to research and practice. First, we contributed to the micro-CSR literature by considering the role of employees' CSR perceptions in dealing with job insecurity. We explored how CSR perceptions moderate the relationship between job insecurity and emotional exhaustion through organizational identification. In this way, we found that CSR has both bright-side and dark-side effects on the negative influences of job insecurity. Second, we disclosed distinctive effects of quantitative and qualitative job insecurity on emotional exhaustion with regard to employees' CSR perception and organizational identification. Our study advances current understanding about the effect of job insecurity and how organizations shape such effects that we identified.

## Literature review and hypotheses

### Relationship among job insecurity, emotional exhaustion, and workplace deviance

Job insecurity is one type of work-related stress, a subjective experience that denotes perceived threats to the job as a whole and its continued existence in the future (4, 18). The individual feels insecure in a job can range from losing one's job ultimately to losing some important features of the job (e.g., wage, promotion opportunities, and work conditions) (19). Accordingly, Hellgren et al. (20) introduced quantitative and qualitative aspects of job insecurity. Quantitative job insecurity is described as concerns about loss of the job itself, whereas qualitative job insecurity is related to perceived threats of losing valuable job features (3, 20). Job insecurity also can be distinguished by cognitive and affective job insecurity. Affective insecurity is associated with emotional state (e.g., concern, worry, and fear) (21), while cognitive insecurity is more likely to relate to job attitudes (e.g., engagement and commitment) (6).

Emotional exhaustion can be defined as “feelings of being emotionally overextended and depleted of one's emotional resources” [(22), p. 399]. From the conservation of resource theory (COR) perspective, it suggested that job insecurity threats an employee's resources and therefore triggers strain in the physical and mental exhaustion (6, 23). Thus, job insecurity is positively related to emotional exhaustion (2, 6, 23).

Workplace deviance refers to purposeful behavior that violates organizational norms and is intended to threaten the wellbeing of an organization, its employees, or both (24). Deviance behaviors can lead directly toward individuals (i.e., interpersonal workplace deviance such as aggression, rudeness, and gossiping), and toward the organization (i.e., organizational workplace deviance such as shirking hours, stealing from a company, and leaving early or arriving late to work) (7, 25, 26). Studies suggested that workplace deviance may occur when employees experience high level of job insecurity (4, 7). For instance, Tian et al. (27) argued that job insecurity brings traumas and makes employees do counter-productive behavior (e.g., workplace deviance). Likewise, Huang et al. (7) suggested that job insecurity triggers moral disengagement, leading to interpersonal and organizational deviance. In addition, emotionally exhausted employees are lack of resources to meet job demands and protect their remaining resources, they will become dissatisfied with their current job and then react by deviant behaviors [e.g., (28–30)].

Based on prior literature about the relationship among job insecurity, emotional exhaustion and workplace deviance, we built on the baseline framework about how job insecurity positively relates to emotional exhaustion, which in turn leads to increased workplace deviance.

### The role of employee CSR perceptions

Employee CSR perceptions refer to the overall perception about which employees view their firms' various CSR activities (14, 17). It captures how employees perceive their firms' CSR efforts, a subjective reaction about firms' objective CSR actions (17, 31). Employee's CSR perceptions can impact employees' emotions, attitudes, and behaviors toward their organizations (14, 17), because CSR caters to employees' deontic needs, i.e., employees not only react to the treatment they themselves received but also to the treatment of others (31). El Akremi et al. (32) further argued “employees as members of a corporation, are concerned about, contribute to, perceive, evaluate, and react to their firm's CSR activities” (p. 621). Thus, higher level of CSR perceptions can improve employees' organizational trust, pride, organizational identification [for a meta-analysis see (33)].

In addition, CSR can be used in an instrumental way to prevent certain employee attitudes and behaviors that harm a firm's performance (e.g., turnover intention, employee cynicism, and workplace gossip) (34, 35). For example, Flammer and Luo (36) revealed that CSR can be used as an employee governance tool to increase employee engagement and counter the possibility of adverse behavior. Schwepker et al. (16) found that organizational ethics (i.e., ethical leadership, ethical climate, and CSR) mitigate work-related stress and promote employee wellbeing.

In this study, we expected that employee CSR perceptions mitigate the effect of job insecurity on emotional exhaustion. Job insecure employees face high level of strain and uncertainty about their job, which can deplete the physical, psychological, and mental energy of employees (37). Their energy and mental resources have been entirely consumed by work (23). At that time, perceived CSR may play a buffering role in decreasing the negative effect of job insecurity on emotional exhaustion. First, CSR activities can improve employees' psychological and physiological wellbeing (16). More responsible firms will cultivate a safer and protective context, provide employees safe working environment, daycare programs, training, and other resources related the job, all can help employees to better adjust their long-term career planning, deal with career shock, and reduce the potential threats to job continuity and stability (2, 38). For one extreme example, when COVID-19 pandemic brings death anxiety to employees, employees perceived internal CSR leaves employees feeling less threatened by the pandemic (39).

Second, employee CSR perceptions reflect firm's objective CSR actions toward inside and outside of organizations, including internal CSR (practices aimed at improving employees' wellbeing, such as employee training, safe working conditions, and daycare programs) and external CSR (practices directed to outside-stakeholders' wellbeing, such as pollution prevention and philanthropy), all can contribute to employees' experienced meaningfulness of work (40, 41) and psychological safety (organizational support) (42, 43). A high level of meaningfulness of work leads employees to define stressors as welcomed challenges that are worthwhile to invest energy to deal with (44), and a high level of psychological safety provides relieved, secure, and calm environment to employees, all of which can reduce the challenges of job insecurity on emotional exhaustion. Taken together, we hypothesized the following:

**Hypothesis 1:** Employee CSR perceptions attenuate the relationship between job insecurity and emotional exhaustion.

Social identity theory suggested that “people tend to categorize themselves and others into social groups to develop a positive self-concept by identifying with groups that enhance their self-esteem” [(45), p. 1725, (46)]. When employees define themselves in terms of the organization, and a feeling of belonging to the group, employees have high level of organizational identification (47). Organizational identification increases employees' work behaviors (e.g., commitment, work engagement and job performance) (45, 48).

We proposed that organizational identification mediates the moderating effect of employees' CSR perceptions on the association between job insecurity and emotional exhaustion. First, scholars found that employees view socially responsible firms as respected and attractive organizations, they have greater organizational pride and commitment to such organizations, which in turn increase organizational identification (13, 33, 49). Second, when employees develop high levels of organizational identification, as members of their organization may tolerate workplace stressors (50, 51). In a similar vein, employees who identify strongly with their organization perceive a high sense of organizational support (52) and organizational trust (13), which can offset the negative consequence of job insecurity [e.g., (3, 10, 53)]. Combined with Hypothesis 1, we inferred that CSR perceptions may increase organizational identification at first, then organizational identification moderates the relationship between job insecurity and emotional exhaustion. Formally, we hypothesized the following:

**Hypothesis 2:** The moderating effect of Employee CSR perceptions on the relationship between job insecurity and emotional exhaustion is explained through organizational identification.

## Methods

### Data and sample

We collected data from one of the biggest computer equipment providers in the middle of China during October 10 in 2020 and May 23 in 2021. This company conducted direct online sales and provided customized computer equipment to individual customers. Firms in the computer industry are experiencing fierce market competition and great pressure to innovate (54), employees in such an industry are valued by key performance indicators (or KPI) and face high level of emotional exhaustion. With the support of the chief executive officer, we obtained all employees' name and job number. We coded each questionnaire with a unique number and contacted corresponding employees to answer the questionnaire. The code is difficult to recognize, which can reduce responders' concern of social desirability bias. We collected data in three-waves. At Time 1, we collected job insecurity, perceived CSR, self-efficacy, and demographic variables. At Time 2, we collected emotional exhaustion and organizational identification. Then, at Time 3, we repeated the same process to collect emotional exhaustion and organizational identification, we further collected workplace deviation. The average gap between the time points was 60 days.

Two hundred fifty-four employees participated in Time 1, which dropped to 211 at Time 2, and 149 at Time 3. Four participants were dropped for the missing values in their demographic information. Thus, our final samples include 145 respondents. 66.90% were males and 33.10% were female. The average age was 28.43 (SD = 5.34), the average tenure was 3.26 years (SD = 1.64), the average working hours in 1 week was 48.79 (SD = 10.19). Regarding education, 3.45% of respondents had a middle school education, 22.76% had a high school or vocational school education, 55.17% had a junior college education, and 18.62% were bachelor's degree holders.

### Measures

All measurement scales were adapted from the existing literature. Using back-translation approach, we translated the English scales to Chinese at first, then translated back into English to ensure equivalence of meanings. All measures were rated on a 5-point Likert scale (1 = strongly disagree to 5 = strongly agree) (see Table 1).

**Table 1:** Confirmatory factor analysis results.

**Variables**	**Measure**	**Factor loadings**
Workplace deviation (55) α = 0.976 CR = 0.975 AVE = 0.796	1. Purposely wasted the employer's materials/supplies.	0.825
	2. Complained about insignificant things at work.	0.818
	3. Told people outside the job what a lousy place you work for.	0.803
	4. Came to work late without permission.	0.910
	5. Stayed home from work and said you were sick when you weren't.	0.945
	6. Insulted someone about their job performance.	0.939
	7. Made fun of someone's personal life.	0.940
	8. Ignored someone at work.	0.945
	9. Started an argument with someone at work.	0.859
	10. Insulted or made fun of someone at work.	0.923
Emotional exhaustion (56) α = 0.948 CR = 0.950 AVE = 0.686	1. I feel emotionally drained from my work.	0.504
	2. I feel used up at the end of the workday.	0.707
	3. I feel fatigued when I get up in the morning and have to face another day on the job.	0.850
	4. Working with people all day is really a strain for me.	0.916
	5. I feel burned out from my work.	0.942
	6. I feel frustrated by my job.	0.898
	7. I feel I'm working too hard on my job.	0.781
	8. Working with people directly puts too much stress on me.	0.891
	9. I feel like I'm at the end of my rope.	0.872
Quantitative job insecurity (18) α = 0.828 CR = 0.828 AVE = 0.548	1. I will likely lose my job very soon and it makes me anxious.	0.671
	2. I am not sure I will be able to keep my job.	0.812
	3. I think I may lose my job in the near future.	0.728
	4. I feel insecure regarding the future of my job.	0.742
Qualitative job insecurity (3) α = 0.890 CR = 0.893 AVE = 0.676	1. I will likely lose a lot of benefits associated with the job.	0.821
	2. I believe that I will get less stimulating work tasks in the future.	0.882
	3. I feel worried about future pay development	0.798
	4. I feel worried about my career development in this organization.	0.785
CSR perceptions (14) α = 0.952 CR = 0.954 AVE = 0.805	1. Our business supports employees' education.	0.909
	2. Flexible company policies enable employees to better coordinate work and personal life.	0.882
	3. Our business gives adequate contributions to charities	0.932
	4. A program is in place to reduce the amount of energy and materials wasted in our business.	0.904
	5. We encourage partnerships with local businesses and schools.	0.856
Organizational identification (57) α = 0.912 CR = 0.916 AVE = 0.735	1. I identify strongly with company.	0.684
	2. I feel a strong sense of membership in company.	0.904
	3. I experience a strong sense of belonging to company.	0.936
	4. The values of company overlap with my own values.	0.882
Self-efficacy (58) α = 0.929 CR = 0.930 AVE = 0.627	1. I will be able to achieve most of the goals that I have set for myself.	0.623
	2. When facing difficult tasks, I am certain that I will accomplish them.	0.797
	3. In general, I think that I can obtain outcomes that are important to me.	0.789
	4. I believe I can succeed at most any endeavor to which I set my mind.	0.836
	5. I will be able to successfully overcome many challenges.	0.880
	6. I am confident that I can perform effectively on many different tasks.	0.801
	7. Compared to other people, I can do most tasks very well.	0.786
	8. Even when things are tough, I can perform quite well.	0.798

α, Cronbach's Alpha; CR, Composite Reliability; AVE, Average Variance Extracted.

#### Workplace deviation

Following prior studies [e.g., (59–61)], we measured workplace deviation using a 10-item scale developed by Spector et al. (55). Qin et al. (60) argued that many deviant behaviors are done privately without others' knowledge, a self-reported measure of workplace deviation can disclose such workplace deviation [e.g., (62, 63)]. A sample item is: “purposely wasted the employer's materials/supplies.” The Cronbach's alpha for this variable was 0.976.

#### Emotional exhaustion

Following prior studies [e.g., (28, 64, 65)], we measured emotional exhaustion using a 9-item scale developed by Maslach and Jackson (56). A sample item is: “I feel emotionally drained from my work.” The Cronbach's alpha for this variable was 0.948.

#### Job insecurity

Hellgren et al. (20) argued that job insecurity could be classified by *qualitative job insecurity* (i.e., treats of losing valued job features) and *quantitative job insecurity* (i.e., subjective assessments regarding the potential loss of the job itself). While most prior empirical studies focused on quantitative job insecurity, in this study we measured both qualitative and quantitative job insecurity separately. Quantitative job insecurity was measured by a 4-item scale developed by De Witte (18). A sample item is: “I will likely lose my job very soon and it make me anxious.” The Cronbach's alpha for this variable was 0.828. Qualitative job insecurity was measured by a 4-item scale adopted from Probst et al. (3). A sample item is: “I feel worried about my career development in this organization.” Cronbach's alpha for this variable was 0.890.

#### Employee CSR perceptions

We measured employee perceived CSR using a 5-item scale developed by Rupp et al. (14). It contains five issues including educational programs for employees, corporate philanthropy, green initiatives, community partnerships, and staff work-life balance programs. A sample item is: “our business supports employees' education.” The Cronbach's alpha for this variable was 0.891.

#### Organizational identification

We measured organizational identification using a 4-item scale developed by Smidts et al. (57). A sample item is: “I identify strongly with the company.” The Cronbach's alpha for this variable was 0.912.

#### Control variables

We controlled for several variables that may impact on firm's emotional exhaustion and workplace deviation (60), including age, gender, education level, tenure, working hours, and self-efficacy. Age was in years; gender was coded as 1 for male and 0 for female; educational level was coded as 1 for middle school, 2 for high school or vocational school, 3 for junior college, and 4 for bachelor's; tenure was in years, working hours in 1 week was in hours. Self-efficacy was measured by 8-items developed by Chen et al. (58). A sample item is: “I will be able to achieve most of the goals that I have set for myself.” The Cronbach's alpha for this variable was 0.929.

### Common method variance

Using self-reported measurement can result in common method variance (CMV) (66), we conducted two ways to ensure CMV was not a problem within our data. First, Harman's (67) one-factor test shows a factor that accounts for 35.93% of the variance. A principle component analysis on all items yielded seven factors that together account for 76.49%. Second, following Podsakoff et al. (66), A single-factor confirmatory factor analysis (CFA) in Mplus 7.0 software shows the model fit deteriorates ($\chi_{\left[902\right]}^{2}$ = 5,009.797, *p* < 0.001, CFI = 0.380, TLI = 0.350, RMSEA = 0.177, SRMR = 0.190). Thus, CMV was less of a concern in this study.

## Results

We first conducted analyses to ensure the reliability and validity of the measures. We conducted CFA in Mplus 7.0 software to evaluate model fit of hypotheses model (see the results from Table 2). It reveals that the 7-factor model provides a good fit ($\chi_{\left[879\right]}^{2}$ = 1,513.644, *p* < 0.001, CFI = 0.904, TLI = 0.897, RMSEA = 0.071, SRMR = 0.053). All scale items loaded on their intended factors significantly. As shown in Table 1, we checked the reliability by using Cronbach's α and the composite reliabilities (CR), all above commonly accepted thresholds. We also examined the convergent validity (average variance extracted, AVE) and discriminant validity. AVE for all variables exceeded the 0.50 benchmark. The discriminant validity shows that the 7-factor model is better than any 6-factor models. We further found that the values of the square root of AVE are higher than the correlations between variables, it reveals that the discriminant validity is appropriate. Overall, these results satisfied the validity and validity.

**Table 2:** Confirmatory factor analysis results for the measures of all variables.

	**χ^2^/df**	**CFI**	**TLI**	**RMSEA**	**SRMR**
Threshold	< 3	≧0.90	≧0.90	≦0.08	≦0.08
7 factor model: Proposed	1.722	0.904	0.897	0.071	0.053
6 factor model	2.047	0.86	0.851	0.085	0.072
1 factor model	5.554	0.380	0.350	0.177	0.190

7 factor model: Hypotheses model.

6 factor model: quantitative job insecurity and qualitative job insecurity combined; We also compared other 6 factor models, we haven't reported results for the space.

1 factor model: all variables combined.

Table 3 presents the means, standard deviations, and correlations among variables. Consistent with prior studies, quantitative and qualitative job insecurity were positively correlated with emotional exhaustion (*r* = 0.265 and 0.215, *p* < 0.01, respectively), emotional exhaustion was positively associated with workplace deviance (*r* = 0.515, *p* < 0.01). Further, quantitative job insecurity was positively related to qualitative job insecurity (*r* = 0.393, *p* < 0.01).

**Table 3:** Descriptive statistics and correlations.

**Variables**	**1**	**2**	**3**	**4**	**5**	**6**	**7**	**8**	**9**	**10**	**11**	**12**
1. WD	*(0.892)*											
2. EE	0.515^**^	*(0.828)*										
3. JI-quant	0.204^*^	0.265^**^	*(0.740)*									
4. JI-qual	0.148^+^	0.215^**^	0.393^**^	*(0.822)*								
5. CSR	−0.351^**^	−0.409^**^	−0.231^**^	−0.163^+^	*(0.897)*							
6. OI	−0.436^**^	−0.573^**^	−0.262^**^	−0.088	0.500^**^	*(0.857)*						
7. Age	−0.068	−0.181^*^	−0.085	0.031	0.073	0.116	–					
8. Gender	0.142^+^	0.086	0.068	0.146^+^	−0.117	−0.092	−0.123	–				
9. Education	0.075	−0.054	0.033	−0.229^**^	−0.100	−0.066	−0.140^+^	0.034	–			
10. Tenure	0.067	0.035	0.020	0.027	−0.122	0.030	0.352^**^	0.163^+^	−0.037	–		
11. WH	0.054	0.108	0.133	0.092	−0.118	−0.148^+^	0.028	0.228^**^	−0.185^*^	0.139^+^	–	
12. SE	−0.220^**^	−0.327^**^	−0.299^**^	−0.228^**^	0.515^**^	0.428^**^	0.196^*^	0.041	−0.015	0.064	−0.143^+^	*(0.792)*
Mean	1.667	2.151	2.740	2.050	4.225	4.079	28.428	0.669	2.890	3.256	48.786	3.792
SD	0.951	0.863	0.869	0.894	0.883	0.750	5.341	0.472	0.737	1.640	10.189	0.693

** *p* < 0.01;

* *p* < 0.05;

+ *p* < 0.10 (two-tailed).

The square root of the AVE values is reported in the parentheses along the diagonal.

WD, workplace deviance; EE, emotional exhaustion; JI-quant, quantitative job insecurity; JI-qual, qualitative job insecurity; CSR, employee CSR perceptions; OI, organizational identification; WH, working hours; SE, self-efficacy.

**Figure 2:**
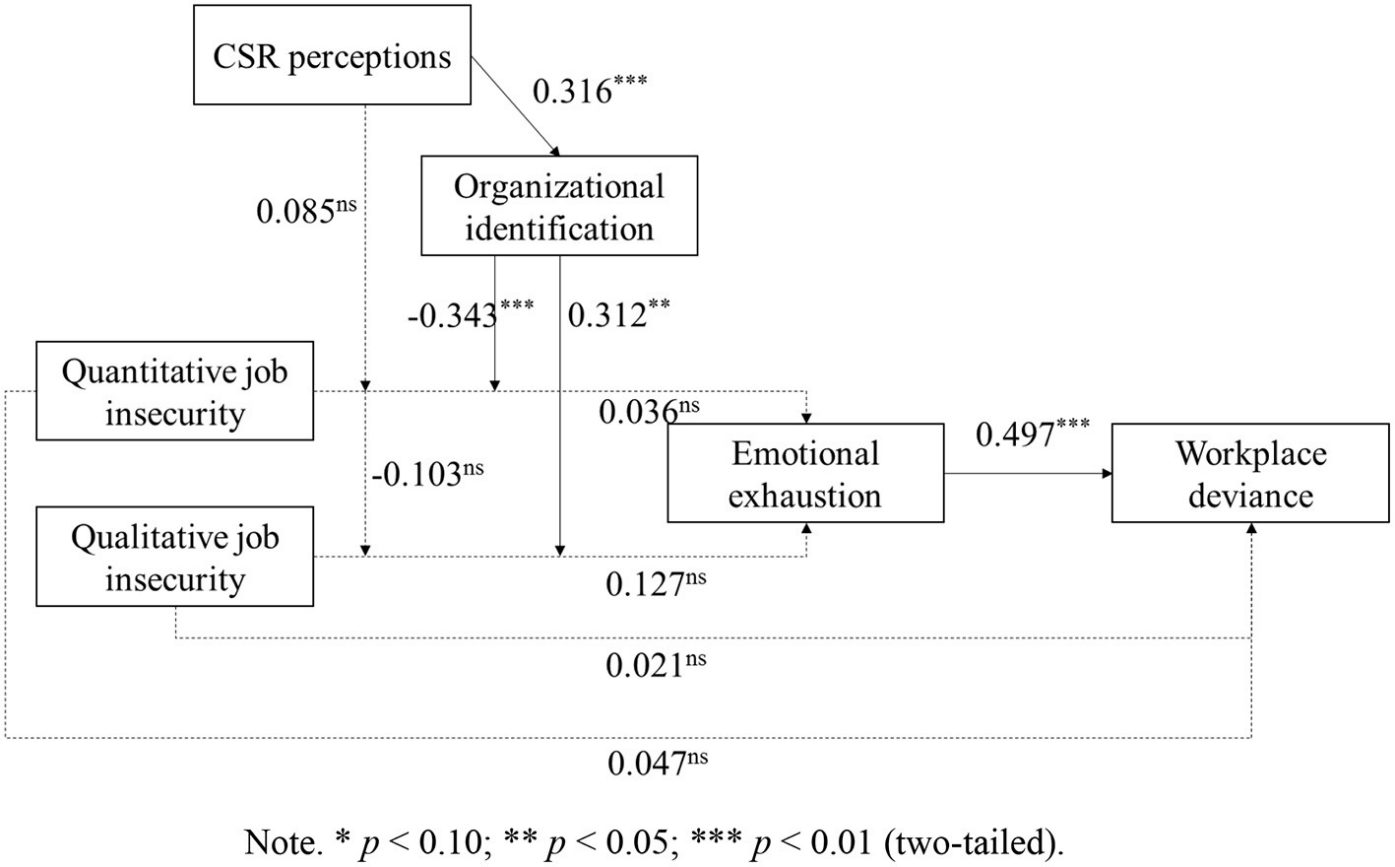
Structural model. *p < 0.10; **p < 0.05; ***p < 0.01 (two-tailed). ns, non significant.

We tested our hypotheses with hierarchical moderated regression in Mplus 7.0. Following Cohen et al. (68), we mean-centered variables to calculate the interactions of job insecurity with CSR perceptions and job insecurity with organizational identification. Hypothesis 1 predicted that CSR perceptions would moderate the association between job insecurity and emotional exhaustion. As Table 4 and Figure 2 shows, we found non-significant interaction effects between quantitative job insecurity and CSR perceptions [β = 0.085, 95% CI (−0.161, 0.267)], and between qualitative job insecurity and CSR perceptions [β = −0.103, 95% CI (−0.265, 0.069)], on emotional exhaustion. Thus, Hypothesis 1 was not supported. It suggests that employee CSR perceptions can't moderate the effect of job insecurity on emotional exhaustion directly.

**Table 4:** Regression results.

	**Emotional exhaustion**		**Workplace deviance**		**OI**	
**Variables**	β	**95% CI**	β	**95% CI**	β	**95% CI**
Constant	3.363	(2.155, 4.476)	0.003	(−1.885, 1.562)	−0.632	(−1.724, 0.443)
**Controls**
Age	−0.028	(−0.051, −0.008)	0.009	(−0.016, 0.035)	0.000	(−0.025, 0.024)
Gender	−0.023	(−0.277, 0.228)	0.179	(−0.147, 0.496)	−0.091	(−0.324, 0.137)
Education	−0.125	(−0.308, 0.061)	0.112	(−0.051, 0.327)	−0.009	(−0.162, 0.147)
Tenure	0.050	(−0.026, 0.111)	0.004	(−0.080, 0.090)	0.037	(−0.039, 0.103)
Working hours	0.000	(−0.011, 0.010)	−0.002	(−0.015, 0.009)	−0.005	(−0.015, 0.005)
Self-efficacy	−0.066	(−0.268, 0.141)	0.002	(−0.261, 0.300)	0.218	(0.032, 0.427)
**Predictors**
Quantitative job insecurity (JI-quant)	0.036	(−0.168, 0.225)	0.047	(−0.166, 0.279)	−0.118	(−0.298, 0.049)
Qualitative job insecurity (JI-qual)	0.127	(−0.118, 0.337)	0.021	(−0.208, 0.241)	0.070	(−0.068, 0.206)
CSR perceptions (CSR)	**−0.175**	**(−0.329**, **−0.033)**	−0.151	(−0.393, 0.057)	**0.316**	**(0.180, 0.458)**
Organizational identification (OI)	**−0.438**	**(−0.601**, **−0.237)**				
JI-quant × CSR	0.085	(−0.161, 0.267)				
JI-quant × OI	**−0.343**	**(−0.553**, **−0.138)**				
JI-qual × CSR	−0.103	(−0.265, 0.069)				
JI-qual × OI	**0.312**	**(0.033, 0.552)**				
Emotional exhaustion			**0.497**	**(0.234, 0.759)**		
**Mediated moderation effect**			**γ**	**95% CI**		
CSR → OI → (JI-quant – emotional exhaustion)			**−0.108**	**(−0.220**, **−0.044)**		
CSR → OI → (JI-qual – emotional exhaustion)			**0.099**	**(0.015, 0.214)**		
*R* ^2^	0.454		0.301		0.319	

*N* = 145; 95% CI represents 95% bias-corrected confidence intervals produced from 5,000 bootstrapped estimates.

The bold values used to make the significant effects more clear.

Hypothesis 2 stated that organization identification mediates the moderation effect of CSR perceptions on the relationship between job insecurity and emotional exhaustion. We completed the test by the suggestion from Edwards and Lambert (69) and other studies [e.g., (70, 71)]. In Table 4, CSR perceptions was significantly related to organizational identification [β = 0.316, 95% CI (0.180, 0.458)], the interaction effect between organizational identification and job insecurity on emotional exhaustion is significant [β = −0.343, 95% CI (−0.553, −0.138); β = 0.312, 95% CI (0.033, 0.552), respectively]. We constructed bias-corrected confidence intervals by drawing 5,000 random samples with replacement from the full sample. The indirect effect results reveal that organizational identification mediated the moderation effect of CSR perception on the relationship between quantitative job insecurity and emotional exhaustion [γ = −0.108, 95% CI (−0.220, −0.044)], and the relationship between qualitative job insecurity and emotional exhaustion [γ = 0.099, 95% CI (0.015, 0.214)], thus supporting for Hypothesis 2.

### Supplement analyses

(1) We combined quantitative and qualitative job insecurity into a unidimensional construct as previous studies have been conducted (2, 7). We repeated our analyses and the results can be seen in Table 5. Consistent with previous study, we found job insecurity positively relates to emotional exhaustion [β = 0.172, 95% CI (0.000, 0.380)], emotional exhaustion positively associates with workplace deviance [β = 0.498, 95% CI (0.225, 0.745)], and emotional exhaustion mediates job insecurity and workplace deviance [γ = 0.086, 95% CI (0.004, 0.258)]. Thus, our base-line model (JI → emotional exhaustion → workplace deviance) was supported. Yet, quite different from the main results in Table 4, we found a non-significant mediated moderation effect [γ = −0.019, 95% CI (−0.099, 0.047)], suggesting that effects differ according to quantitative vs. qualitative job insecurity.
(2) We also measured emotional exhaustion at Time 3, so we examined the lagged effect of moderating effect of employee CSR perceptions on the relationship between job insecurity and emotional exhaustion. The Cronbach's alpha for emotional exhaustion was 0.948. We repeated our analyses and found similar results (see detail from Table 6).

**Table 5:** Supplement analysis 1: results of unidimensional construct of job insecurity.

	**Emotional exhaustion**		**Workplace deviance**		**OI**	
**Variables**	β	**95% CI**	β	**95% CI**	β	**95% CI**
Constant	3.080	(1.840, 4.272)	−0.008	(−1.761, 1.521)	−0.560	(−1.686, 0.549)
**Controls**
Age	−0.026	(−0.046, −0.005)	0.008	(−0.017, 0.035)	0.002	(−0.024, 0.027)
Gender	−0.037	(−0.303, 0.221)	0.176	(−0.160, 0.506)	−0.067	(−0.305, 0.165)
Education	−0.117	(−0.282, 0.028)	0.116	(−0.044, 0.308)	−0.040	(−0.185, 0.111)
Tenure	0.045	(−0.035, 0.112)	0.005	(−0.078, 0.094)	0.034	(−0.041, 0.100)
Working hours	−0.001	(−0.013, 0.009)	−0.002	(−0.015, 0.009)	−0.006	(−0.016, 0.004)
Self-efficacy	0.022	(−0.211, 0.284)	0.002	(−0.266, 0.287)	0.220	(0.035, 0.426)
**Predictors**
Job insecurity (JI)	**0.172**	**(0.000, 0.380)**	0.067	(−0.133, 0.280)	−0.047	(−0.209, 0.095)
CSR perceptions (CSR)	−0.139	(−0.293 0.012)	−0.151	(−0.393, 0.052)	**0.318**	**(0.182, 0.466)**
Organizational identification (OI)	**−0.538**	**(−0.709**, **−0.354)**				
JI × CSR	0.033	(−0.152, 0.220)				
JI × OI	−0.059	(−0.295, 0.149)				
Emotional exhaustion			**0.498**	**(0.225, 0.745)**		
**Mediated moderation effect**			**γ**	**95% CI**		
CSR → OI → (JI – emotional exhaustion)			−0.019	(−0.099, 0.047)		
*R* ^2^	0.398		0.307		0.306	

*N* = 145; 95% CI represents 95% bias-corrected confidence intervals produced from 5,000 bootstrapped estimates.

The bold values used to make the significant effects more clear.

**Table 6:** Supplement analysis 2: results of emotional exhaustion at Time 3.

	**Emotional exhaustion**		**Workplace deviance**		**OI**	
**Variables**	β	**95% CI**	β	**95% CI**	β	**95% CI**
Constant	5.071	(3.565, 6.527)	−0.743	(−2.500, 0.923)	−0.602	(−1.804, 0.508)
**Controls**
Age	−0.013	(−0.036, 0.015)	−0.006	(−0.034, 0.017)	0.004	(−0.002, 0.030)
Gender	0.148	(−0.169, 0.490)	0.058	(−0.261, 0.379)	−0.125	(−0.350, 0.108)
Education	−0.341	(−0.539, −0.132)	0.174	(−0.060, 0.403)	0.049	(−0.132, 0.237)
Tenure	0.011	(−0.076, 0.089)	0.032	(−0.050, 0.120)	0.028	(−0.050, 0.092)
Working hours	−0.016	(−0.035, −0.002)	0.008	(−0.004, 0.022)	−0.004	(−0.015, 0.005)
Self-efficacy	−0.237	(−0.523, −0.005)	0.084	(−0.166, 0.352)	0.145	(−0.066, 0.361)
**Predictors**
Quantitative job insecurity (JI-quant)	0.149	(−0.073, 0.340)	0.058	(−0.148, 0.270)	−0.151	(−0.332, 0.014)
Qualitative job insecurity (JI-qual)	−0.009	(−0.234, 0.209)	0.084	(−0.129, 0.280)	0.056	(−0.088, 0.178)
CSR perceptions (CSR)	−0.190	(−0.418, 0.008)	**−0.189**	**(−0.418**, **−0.002)**	**0.315**	**(0.192, 0.470)**
Organizational identification (OI)	−0.063	(−0.267, 0.187)				
JI-quant × CSR	−0.146	(−0.438, 0.109)				
JI-quant × OI	**−0.307**	**(−0.551**, **−0.082)**				
JI-qual × CSR	0.050	(−0.227, 0.286)				
JI-qual × OI	0.289	(−0.005, 0.571)				
Emotional exhaustion			**0.555**	**(0.327, 0.788)**		
**Mediated moderation effect**			**γ**	***95% CI***		
CSR → OI → (JI-quant – emotional exhaustion)			**−0.097**	**(−0.215**, **−0.031)**		
CSR → OI → (JI-qual – emotional exhaustion)			**0.091**	**(0.005, 0.218)**		
*R* ^2^	0.273		0.369		0.289	

*N* = 143; 95% CI represents 95% bias-corrected confidence intervals produced from 5,000 bootstrapped estimates.

The bold values used to make the significant effects more clear.

## Discussion

Job insecurity is one of top concerns in the current workforce. Integrating micro-CSR literature and social identify theory, we hypothesized and tested whether employee perceived CSR plays a buffer role to the negative effect of job insecurity. We found that when job insecurity was considered as a unidimensional construct, the moderation role of employee CSR perceptions was not supported. In contrast, a nuance perspective shows that employee CSR perceptions moderate the link between quantitative and qualitative job insecurity and emotional exhaustion indirectly through organizational identification.

More interestingly, employee CSR perceptions alleviate (exacerbate) the negative relationship between quantitative (qualitative) job insecurity and emotional exhaustion *via* organization identification. Quantitative job insecurity implies a loss of job itself and qualitative job insecurity implies a loss of valued job features, thus the former concerns the frustration of a general need (security need) and the later concerns the frustration of a particular need (growth needs) (72, 73). Organizational identification generated by CSR will satisfy several individual needs including safety, affiliation, and uncertainty reduction (74, 75), which all relate to security need instead of growth need. Lawrence and Kacmar (76), further, argued that facing job insecurity the high level of attachment and embeddedness to organization will do harm to regulate employees' feelings and redirect their attention to new job opportunities. Therefore, CSR and organizational identification play different roles with respect to quantitative vs. qualitative job insecurity.

### Theoretical implications

This study makes two contributions to the literature. First, to the micro-CSR literature, this study is one of the first studies that considered employee perceived CSR in dealing with the negative effect of job insecurity. CSR cannot be a panacea, it has both bright-side and dark-side effects (41, 77, 78). While, mostly prior studies considered only the desirable or the undesirable results of CSR separately [e.g., (79–81)], this study revealed both effects exist. Furthermore, we introduced organizational identification as a mechanism for explaining the moderating effects of CSR perceptions on the relationship between job insecurity and emotional exhaustion. Thus, this study provides a deeper understanding about how employees' CSR perceptions react to the insecure job environment.

Second, to the job insecurity research, we responded Shoss's (4) suggestion that “future work should examine whether effects differ according to quantitative vs. qualitative job insecurity” (p. 1934) [see also from Jiang and Lavaysse (6)]. A unidimensional construct of job insecurity leads to emotional exhaustion and further to workplace deviance, but such relationship become non-significant when we divided job insecurity into quantitative and qualitative job insecurity. Moreover, quantitative and qualitative job insecurity generate distinctive effects on emotional exhaustion when considering employees' CSR perceptions and organizational identification. Thus, our study advances the current understanding about the effect of job insecurity and how to shape such effects.

### Managerial implications

Based on our empirical results, this study is also important for managers. Managers should pay attention to the risk of job insecurity to employees' emotional exhaustion and workplace deviance. When facing job insecure employees, managers should distinguish job insecurity from quantitative vs. qualitative job insecurity at first. If employees face quantitative job insecurity, managers can proactively engage in CSR and use communication efforts to enhance CSR awareness to employees. Our results suggest that CSR perceptions generate organizational identification, which can mitigate the negative effect of quantitative job insecurity. While, if employees face qualitative job insecurity, of course engaging in CSR activities is good, but communicating CSR to employees may produce counterproductive effects.

### Limitations and future research directions

This study has several limitations that should be addressed in the future. First, we found one way through which employee CSR perceptions moderate the relationship between job insecurity and emotional exhaustion, prior micro-CSR studies considered multiple ways through which employees perceived CSR leads to organizational and individual outcomes, such as organizational trust, organizational pride, psychological safety, and meaningfulness of work [e.g., (17, 78, 82)]. Future studies can consider those mechanisms related to employee CSR perceptions and their effects to alleviate or exacerbate the negative impact of job insecurity.

Second, future studies might consider other outcomes that are derived from job insecurity. For example, job insecurity yields moral disengagement (3, 7), whether employee CSR perceptions play a role in the job insecurity-moral disengagement link.

Third, although this study used three-wave data collection to minimize CMV problems and disentangle the causal order of our variables, we recommended further research using experimental and field studies to obtain more substantial causal effects.

## Data availability statement

The original contributions presented in the study are included in the article/supplementary material, further inquiries can be directed to the corresponding author.

## Ethics statement

The studies involving human participants were reviewed and approved by Hubei University. The patients/participants provided their written informed consent to participate in this study.

## Author contributions

XJ has developed the research model, analyzed the data, and co-drafted the manuscript. SL has helped to collect the data and co-drafted the manuscript. WY has edited the manuscript. All authors contributed to the article and approved the submitted version.

## Funding

This work was funded by the Young Scientists Fund of the National Natural Science Foundation of China (Grant Nos. 71902056 and 71802073) and Young Scientists Foundation of Innovative Research Team in Hubei University (HBQN0103).

## Conflict of interest

The authors declare that the research was conducted in the absence of any commercial or financial relationships that could be construed as a potential conflict of interest.

## Publisher's note

All claims expressed in this article are solely those of the authors and do not necessarily represent those of their affiliated organizations, or those of the publisher, the editors and the reviewers. Any product that may be evaluated in this article, or claim that may be made by its manufacturer, is not guaranteed or endorsed by the publisher.
